# Corin—The Early Marker of Preeclampsia in Pregestational Diabetes Mellitus

**DOI:** 10.3390/jcm12010061

**Published:** 2022-12-21

**Authors:** Daniel Boroń, Jakub Kornacki, Paweł Gutaj, Urszula Mantaj, Przemysław Wirstlein, Ewa Wender-Ozegowska

**Affiliations:** 1 Department of Reproduction, Poznań University of Medical Sciences, 61-701 Poznan, Poland; 2 PUMS Doctoral School, 61-701 Poznan, Poland

**Keywords:** corin, preeclampsia, pregestational diabetes

## Abstract

Preeclampsia (PE) is one of the leading causes of mortality and morbidity in pregnant women. Pregestational diabetes (PGDM) patients are prone to vascular complications and preeclampsia, whereas vascular exposure to hyperglycemia induces inflammation, vascular remodeling, and arterial stiffness. Corin is a serine protease, converting inactive pro-atrial natriuretic peptide (pro-ANP) into an active form. It also promotes salt and water excretion by activating atrial natriuretic peptide (ANP), and significantly increases trophoblast invasion. The study aimed to determine whether corin may be a predictor of PE in a high-risk group—women with long-term PGDM. The nested case-control prospective study involved 63 patients with long-term pregestational type 1 diabetes (PGDM). In total, 17 patients developed preeclampsia (the study group), whereas 43 patients without PE constituted the control group. To assess corin concentration, blood samples were collected at two time points: between 18th–22nd week of gestation and 28th–32nd week of gestation. PE patients presented significantly higher mid-gestation corin levels, urine protein loss in each trimester, serum creatinine in the third trimester, and lower creatinine clearance in the third trimester. The results of our study indicate that serum corin assessment may play a role in predicting preeclampsia. Thus, it may be included in the PE risk calculator, initially in high-risk groups, such as patients with PGDM.

## 1. Introduction

Preeclampsia constitutes one of the leading causes of mortality and morbidity in pregnant women. Moreover, it remains a challenge for the public health, particularly in high-income countries. In fact, approximately 10–15% of pregnancy-associated maternal deaths are due to complications related to preeclampsia (PE) [1,2,3]. Nevertheless, effective diagnostic tools to predict the development of preeclampsia remain scarce, as well as the methods of treating it, other than the symptomatic treatment (anti-hypertensive drugs) or timing the delivery, which is the only effective PE treatment.

It is suggested that PE results from impaired cytotrophoblast invasion [4]. This, in turn, leads to early subclinical malfunction of the placenta and is transferred to the maternal uterine arteries, resulting in an increased pulsatility index (PI) [5]. In order to assess PE risk, uterine artery PI is determined in the first trimester scan [3]. The association between the maternal arterial stiffness and PE development has been shown both before and during pregnancy [6,7]. Nevertheless, patients with only minor vascular changes, which may lead to an increased arterial stiffness, frequently experience no symptoms prior to the pregnancy and are usually unaware of their condition. In contrast, patients with pregestational diabetes (PGDM), particularly long-term PGDM, are a group particularly susceptible to vascular complications and preeclampsia. It is worth bearing in mind that vascular exposure to hyperglycemia induces inflammation, vascular remodeling, and arterial stiffness [8,9], whereas local inflammation affects the intima-media complex, increasing its thickness and pulsatility index [10,11]. Hence, it is essential to identify a group of patients at risk of developing PE, not only because of pregnancy complications and different follow up in the course of pregnancy, but also due to the increased risk of cardiovascular disease (CVD) and death associated with an acute cardiovascular event [12,13,14].

Natriuretic peptides, involved in the prevention of hypertension, increase urine production; thus, reducing intravascular volume. In view of the reduced a intravascular volume, the initial thesis assumed that women with preeclampsia would present lower serum levels of natriuretic peptides [15]. However, the published studies indicate that patients with preeclampsia show significantly higher levels of natriuretic peptides due to the increased peripheral vascular resistance [16]. Nonetheless, the prediction value based on natriuretic peptide levels is limited. Most publications show increased levels of natriuretic peptides in patients with PE, although the concentration is unremarkable in patients prior to PE development [17].

Corin is a serine protease found in the heart, converting inactive pro-atrial natriuretic peptide (pro-ANP) into an active form [18]. It promotes salt and water excretion by atrial natriuretic peptide (ANP) activation. Animal models show that a lack of corin leads to salt-sensitive hypertension, gestational cardiomyopathy, and preeclampsia in mice [19,20]. Interestingly, decreased corin plasma levels in humans were reported in patients with heart failure and corin was suggested as a biochemical marker of cardiovascular disease [21,22,23]. Similarly, reduced corin renal expression was observed among patients with glomerular diseases associated with salt retention [24].

During pregnancy, particularly in late pregnancy, corin serum concentration increases compared to the pre-pregnancy level and then, returns to the basal level following delivery. It is of note that gestational corin elevation is more significant among women with preeclampsia and gestational hypertension [25]. This, in turn, corresponds with how the increased afterload impacts maternal circulation during normotensive pregnancy and pregnancy complicated by the increased peripheral/placental resistance, causing hypertensive disorders of pregnancy. Moreover, corin expression was found in the uteri of pregnant women, and its expression was significantly lower among patients with PE [26]. Animal models and human observations support the hypothesis that corin significantly promotes trophoblast invasion and spiral artery remodeling [27]. In fact, an elevated corin serum level was found in pregnancies complicated by preeclampsia and fetal growth restriction (FGR). However, corin mRNA expression was not increased in either of these complications. Therefore, the upregulation of corin may not only be associated with hypertension, but may also play a role in the common pathway of PE and FGR pathogenesis [28].

The presented study aimed to determine whether corin may be effective in predicting preeclampsia, fetal growth restriction, and gestational hypertension in a high-risk group—women with long-term pregestational diabetes. The other goal was to identify the determinants of elevated corin levels in PGDM patients.

## 2. Materials and Methods

### 2.1. Patients

This prospective study involved 63 patients in a singleton pregnancy with long-term pregestational type 1 diabetes (PGDM). Recruitment of patients was conducted at the Department of Reproduction at Poznań University of Medical Sciences, a tertiary-care center specializing in PGDM treatment, between April 2019 and July 2022. Patients were all Caucasian and received a standard pregnancy care for diabetes, as recommended by the Polish Diabetes Association and Polish Gynecological Society, targeting a fasting glucose level of 3.8–5.0 mmol/L, 1-h postprandial glucose below 7.0 mmol/L, and glycated hemoglobin (HbA_1c_) below 6.0% (42 mmol/mol) [29]. All the women were treated with intensive insulin therapy, either with multiple daily insulin (MDI) injections or continuous subcutaneous insulin infusion (CSII). According to the Polish recommendations, all the patients received 150 mg of aspirin daily from the 12th–the 36th week of gestation as a form of preventing preeclampsia [29].

In all, 63 women met the inclusion criteria: singleton pregnancy, long-term type 1 diabetes (class C, D, F, R, according to the White classification [30]), no history of preeclampsia or gestational hypertension. In total, 3 patients were excluded from the study due to the withdrawal of consent (*n* = 2) and spontaneous abortion (*n* = 1). No major fetal anatomical abnormality or aneuploidy was diagnosed in these pregnancies.

In order to diagnose preeclampsia, the ISSHP (International Society for the Study of Hypertension in Pregnancy) criteria [31] were used, with adjustments for patients with diabetes according to Kornacki et al. [32]: in patients who had not been previously diagnosed with chronic hypertension (*n* = 48) [31]—systolic blood pressure (BP) ≥ 140 mmHg or diastolic BP ≥ 90 mmHg on two specific instants which occurred for the first time after 20 weeks of gestation, and one of the following complications with the onset in the second half of pregnancy: (1) proteinuria (≥300 mg/24 h or >100% increase in proteinuria in proteinuric patients); (2) serum creatinine > 1 mg/dL (>90 μmol/L) or >50% increase in serum creatinine within 7 days; (3) elevation of transaminase levels > 40 IU/L; (4) neurological complications (eclampsia, altered mental status, blindness, stroke, clonus, severe headache, persistent visual scotomata); (5) hematological complications (thrombocytopenia <150 G/L, disseminated intravascular coagulation, hemolysis); and (6) uteroplacental dysfunction (fetal growth restriction, Doppler indices of placental insufficiency [33]). Moreover, in patients with chronic hypertension (*n* = 12), PE was diagnosed following the onset of severe hypertension (systolic BP > 160 mmHg or diastolic BP > 110 mmHg) after 20 weeks of gestation or the need to increase treatment to maintain BP < 160/110 mmHg in the second half of pregnancy. These had to be accompanied by at least one of the abovementioned criteria for patients without previous chronic hypertension, except uteroplacental dysfunction, according to ISSHP [31]. Fetal growth restriction (FGR) was diagnosed according to the Delphi consensus criteria [33].

### 2.2. Monitoring of Laboratory and Clinical Measurements

The following laboratory tests were performed in each trimester in all the patients: (1) glycated hemoglobin (HbA1c), (2) daily urine protein loss, (3) serum creatinine, (4) creatinine clearance, and (5) concentration of serum triglycerides (TG). In terms of the clinical characteristics, they comprised maternal height, pregestational weight, body weight just before the delivery, weight gain, Doppler ultrasound examination, insulin intake, and the data concerning the delivery (method, timing, complications, neonatal results) presented in Table 1.

Blood samples for all the routine analyses were collected following overnight fasting and immediately transported to the accredited university hospital laboratory, with the ISO 9000 quality management certification. HbA1c in whole blood was determined using the turbidimetric inhibition immunoassay (TINIA), Tina-quant Hemoglobin A1c II test in a Cobas c311 analyzer (Roche Diagnostics). The normal range for a non-pregnant population amounts to 29–42 mmol/mol (4.8–6.0%). In the presented study, three HbA1c values were used: the first was measured in the first trimester or at first admission, the second was measured in the second trimester between 18 + 0 and 22 + 0 gestational weeks, and the third was measured prior to the delivery (up to six days before). The mean value was used if two HbA1c values were measured in the second trimester.

Blood samples for corin concentration assessment were collected at two time points: between the 18th–22nd and the 28th–32nd week of gestation. In total, 119 blood samples were collected and maternal-corin serum levels were measured in all cases. A nested case-control study was performed, and all women were divided into the PE group (*n* = 13) and controls (*n* = 47) based on pregnancy outcome.

In order to determine corin concentration, 7.5 mL of venous blood was collected from patients with PE and from the control group. After centrifugation (2000× *g*) of the blood samples, the obtained serum was frozen at −20 °C for further assessment. Corin concentrations were determined by immuno-enzymatic tests (enzyme-linked immunosorbent assay [ELISA] kit) procured from Develop (DLR-CRN-Hu; Wuxi Donglin Sci & Tech Development Co., Ltd. Jangsu, China). The assays were performed according to the manufacturer’s instructions. Plate reading was conducted using an MRX reader (Dynex Technologies, Chantilly, VA, USA) at λ = 450 nm, with corrections at 570 nm.

Written informed consent was obtained from each patient prior to enrolment and blood sampling. The study was approved by the Bioethics Committee at Poznań University of Medical Sciences and conducted in accordance with the Declaration of Helsinki (No. 291/21). The Bioethics Committee at Poznan University of Medical Sciences reviewed the study protocol and confirmed that the conducted research was not a clinical trial.

### 2.3. Statistical Analysis

The analysis was conducted in the PQStat 1.8.4 program (PQStat software, Poznan, Poland). The Lilliefors test was applied for the verification of normality and compared the groups using the *t*-student test for data that followed a normal distribution. To compare the data that did not follow a normal distribution, the Mann–Whitney test was used; and for categorical variables, the chi-square test with Yates modification was applied. Multivariate multiple regression analysis was performed to determine factors affecting corin serum concentration.

## 3. Results

In the studied population, 17 patients developed preeclampsia, whereas 43 women without PE constituted the control group. There were no significant differences between the preeclampsia and the control group in terms of the maternal age, height, BMI (neither pregestational, nor at term), weight gain during pregnancy, parity, diabetes duration, and presence of vascular complications on admission. Vascular complications in PGDM included nephropathy, retinopathy, peripheral neuropathy, and major vascular events described in the patients’ history. The number of patients with chronic hypertension and fetal growth restriction was higher in the PE group (Table 1).

**Figure 1:**
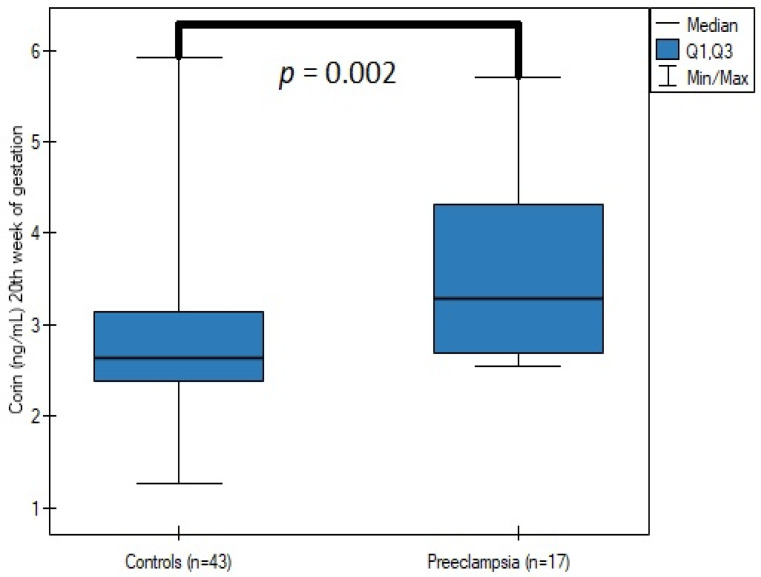
The comparison of corin serum concentrations at the 20th week of gestation between the patients with and without preeclampsia.

Patients with preeclampsia presented significantly higher mid-gestation corin levels, urine protein loss in each trimester, as well as serum creatinine in the third trimester, and lower creatinine clearance in the third trimester (Table 2) (Figure 1). Gestational age at delivery and neonate weight were lower in the preeclampsia group. However, the ratio of cesarean and emergency cesarean sections were unremarkable in the two groups.

The duration of diabetes (*p* = 0.81), vascular complications (*p* = 0.22), in each trimester additionally: HbA1c (*p* = 0.47; *p* = 0.97; *p* = 0.81, respectively), triglycerides (*p* = 0.49; *p* = 0.46; *p* = 0.21, respectively), protein loss within 24 h of urine collection (*p* = 0.74; *p* = 0.85; *p* = 0.67, respectively), creatinine clearance (*p* = 0.19; *p* = 0.59; *p* = 0.5, respectively), and serum creatinine concentration (*p* = 0.73; *p* = 0.94; *p* = 0.82 respectively) were considered as the potential determinants of corin concentration, although none of the above affected protease concentration. Maternal parameters, such as height (*p* = 0.13), weight (*p* = 0.99), pregestational BMI (*p* = 0.51), weight gain (*p* = 0.99), insulin intake (*p* = 0.21), and parity (*p* = 0.69) also did not significantly impact corin concentration.

## 4. Discussion

**Figure 2:**
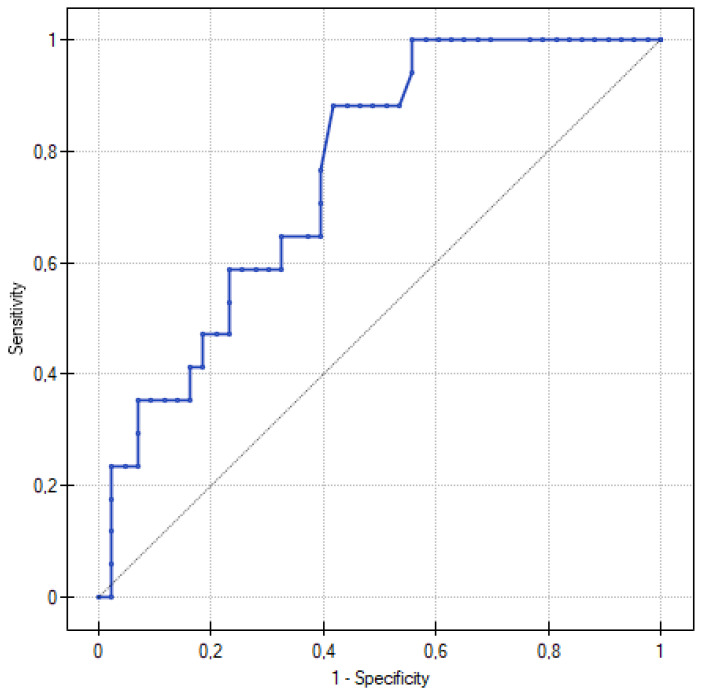
ROC curve for predicting preeclampsia according to corin serum levels in 20th week of gestation. At a corin cut-off value of 2.676 ng/mL, the sensitivity was 88.24% and the specificity was 58.14%, with an AUC = 0.760 (95% CI, 0.637–0.883).

Our results demonstrate the role of corin in the early stages of the pathogenesis of preeclampsia, and indicate the potential use of corin as a biomarker of impaired placental function and trophoblast invasion in patients with pregestational diabetes. The increased corin serum concentration was associated with a higher PE incidence (*p* = 0.002). Moreover, our study indicated a potential role of corin in preeclampsia screening in patients with PGDM. Corin sensitivity and specificity at a cut-off value of 2.676 ng/mL were 88.24% and 58.14%, with an AUC = 0.760 (95% CI, 0.637–0.883) (Figure 2). Additionally, all the parameters considered as the potential determinants of corin concentration did not show statistical significance. The aforementioned results reduce the potential bias created by maternal obesity or preexisting proteinuria, which frequently challenges preeclampsia diagnosis in the day-to-day clinical practice.

Recent studies have showed a correlation between corin serum levels and preeclampsia [19,27,28,34]. Furthermore, the data indicate that serum corin measurements may be applicable not only in terms of detecting preeclampsia itself, but also in predicting it [35]. Corin serum levels may be affected by cardiac expression, which activates natriuretic peptides reducing blood pressure (BP) by means of increasing salt excretion and urine production. Corin expression was observed in the uterus during pregnancy, which, in turn, was related to the fact that uterine expression affected corin serum levels due to the direct contact of syncytiotrophoblast with maternal circulation [36]. Therefore, potentially, the changes in uterine corin expression may participate in the pathomechanism of preeclampsia, whereas cardiac expression of soluble corin may be interpreted as a response to the increased peripheral vascular resistance. Additionally, corin is a transmembrane serine protease, and it was demonstrated that the soluble form found in serum showed the same activity as the membrane-bound form [37].

In our study, the patients and control groups were relatively homogenous. Nevertheless, certain differences in fetal weight, chronic hypertension, and gestational age at delivery in patients with preeclampsia were expected. In fact, placental dysfunction leading to PE development impairs fetal growth and may result in earlier induction of labor or planned caesarian section, as recommended by international and Polish guidelines [38,39].

The role of corin in the pathogenesis of PE remains unclear. Corin expression was detected in the uteri of pregnant women, and by means of activating ANP, trophoblast invasion and spiral arteries remodeling were promoted [26]. Moreover, knocking out corin and ANP genes in the murine model led to salt-sensitive hypertension, preeclampsia, and cardiac hypertrophy [40,41,42]. Hyperinsulinemia in patients with type 2 diabetes is a different risk factor for lower corin expression and immunoreactivity in the placenta, which may also participate in the impaired trophoblast invasion [43]. Mutations in the corin gene were found in humans, more frequently in black individuals; and when present, they significantly increase the risk of hypertension, due to the impaired natriuretic peptide BP regulation [44,45].

Zaki et al. showed that patients with increased blood pressure presented higher corin and natriuretic peptide serum levels [46]. The assumption in our study was that the increased soluble corin levels in patients who subsequently developed preeclampsia may be used as a marker of subclinical increase of peripheral resistance due to the impaired spiral artery remodeling. This hypothesis is supported by the study assessing mid-pregnancy levels of soluble corin and its elevation in patients developing hypertensive disorders in pregnancy [47]. However, Khalil et al., in their longitudinal observation of corin levels throughout pregnancy, presented decreased corin levels until the 20th week of gestation in PE patients, as compared to the controls, as well as increased corin levels in the late second and in the third trimester [34]. Our results demonstrate that in patients with pregestational diabetes, vascular changes are present before conception, and may accelerate placental insufficiency and its impact on the peripheral resistance, leading to earlier corin overexpression aimed to maintain normal blood pressure.

Interestingly, adding plasma-soluble corin to the preeclampsia prediction model improved its effectiveness [35,48]. A combination of corin with the currently used sFlt-1/PLGF ratio may be an ideal marker with respect to predicting preeclampsia, presented by Liu et al., as a marker with the highest AUC in ROC curves analysis comparing different methods [48]. Moreover, our results advocate the inclusion of corin to the standard preeclampsia screening [3]. Patients with pregestational diabetes frequently present with subclinical vascular complications and are particularly at risk of developing hypertensive disorders in pregnancy. Therefore, in view of our research, the incorporation of corin in PGDM patients for PE screening as the most beneficial, providing a greater risk stratification and a better follow-up model, simultaneously reducing the number of complications.

The strengths of this research include the number of pregnant women with pregestational diabetes enrolled for the purpose of the nested case-control study, as well as the homogeneity of the studied groups. In contrast, the study’s limitations comprise the still undetermined pathomechanisms of PE development, as well as the failure to differentiate the serum corin fractions based on the origin of its expression. An additionally increased number of participants would provide stronger evidence for corin assessment in PE prediction. Hopefully, further studies will provide data sufficient to implement corin assessment in clinical practice.

## 5. Conclusions

As the obtained results demonstrate, the assessment of corin serum concentration may play a role in the prediction of preeclampsia. Possibly, it may be included in the PE risk calculator, although initially only in high-risk patient groups, such as patients with PGDM. Further studies on larger populations are essential to establish the most effective protocol for determining the PE risk by means of comparing its efficiency using new biomarkers, such as corin, with the currently used ones; e.g., sFLT-1/PlGF.

## Figures and Tables

**Table 1:** Maternal characteristics in PE patients and the control group.

Parameter	Controls (*n* = 43)	Preeclampsia (*n* = 17)	*p*
Maternal age	30.47 ± 5.67	32 ± 6.24	0.36
Maternal height (m)	1.65 ± 0.077	1.63 ± 0.055	0.44
Maternal bmi at admission (kg/m^2^)	24.28 ± 6.25	24.85 ± 3.88	0.72
Maternal bmi at delivery (kg/m^2^)	27.53 (25.21–31.06)	28.42 (25.8–30.86)	0.74
Weight gain (kg)	10.6 (7.3–15)	9 (6–14)	0.72
Nulliparous	26 (60.47%)	13 (76.47%)	0.38
Vascular complications at admission	14 (32.56%)	10 (58.82%)	0.11
Age at diabetes diagnosis (years)	9 (7–12.5)	11 (9–14)	0.18
Diabetes duration (years)	20 (16–23)	22 (18–23)	0.56
Treatment with the insulin pump	37 (86.05%)	13 (76.47%)	0.37
Chronic hypertension	4 (9.3%)	8 (47.06%)	0.001
Fetal FGR	1 (2.38%)	4 (23.53%)	0.008
Gestational age at delivery (weeks)	38 (37–38)	35 (33–37)	0.00003
Newborns’weight	3348.3 ± 524.9	2591.7 ± 907.3	0.0002
Cesarean section	34 (85%)	17 (100%)	0.09
Emergency cesarian section	8 (23.53%)	6 (35.29%)	0.37

**Table 2:** Differences in biochemical parameters between PE patients and the control group.

Parameter	Controls (*n* = 43)	Preeclampsia (*n* = 17)	*p*
Corin—18th–22nd week of gestation (ng/mL)	2.64 (2.382–3.142)	3.286 (2.684–4.31)	0.002
Corin—28th–32nd week of gestation (ng/mL)	2.93 (2.652–4.174)	2.754(2.668–3.537)	0.55
Hba1c—I trimester (%)	6.50 (6.09–7.31)	6.61 (6.29–7.27)	0.46
Hba1c—II trimester (%)	5.66 ± 0.65	5.99 ± 0.95	0.13
Hba1c—III trimester (%)	5.88 ± 0.61	6.09 ± 0.77	0.27
Triglycerides— I trimester (mg/dL)	64.6 (52.75–85.35)	65.7 (46.6–82.6)	0.84
Triglycerides— II trimester (mg/dL)	132.6 (107.2–158.1)	117.2 (102.5–178.1)	0.94
Triglycerides—III trimester (mg/dL)	280.61 ± 86.41	241.71 ± 51.43	0.09
Proteinuria—I trimester (g/24 h)	0.18 (0.14–0.21)	0.3 (0.2–0.73)	0.003
Proteinuria—II trimester (g/24 h)	0.16 (0.14–0.22)	0.34 (0.15-0.91)	0.02
Proteinuria—III trimester (g/24 h))	0.22 (0.16–0.31)	0.78 (0.38–1.82)	0.000001
Creatinine clearance—I trimester (mL/min)	126.79 ± 41.24	130.59 ± 53.64	0.77
Creatinine clearance—II trimester (mL/min)	138.74 ± 43.15	112.01 ± 53.7	0.054
Creatinine clearance—III trimester (mL/min)	127.87 ± 37.64	99.26 ± 41.8	0.01
Serum creatinine—I trimester (mg/dL)	0.57 (0.52–0.62)	0.62 (0.52–0.79)	0.34
Serum creatinine—II trimester (mg/dL)	0.595 (0.49–0.62)	0.62 (0.49–0.72)	0.13
Serum creatinine—III trimester (mg/dL)	0.6 (0.52–0.69)	0.7 (0.64–0.81)	0.007

## Data Availability

All the raw data are on the clinical server, available upon request.
